# Laser Induced Anchoring of Nickel Oxide Nanoparticles on Polymeric Graphitic Carbon Nitride Sheets Using Pulsed Laser Ablation for Efficient Water Splitting under Visible Light

**DOI:** 10.3390/nano10061098

**Published:** 2020-06-02

**Authors:** Umair Baig, Abuzar Khan, Mohammad A. Gondal, Mohamed A. Dastageer, Wail S. Falath

**Affiliations:** 1 Center of Research Excellence in Desalination & Water Treatment and Center for Environment and Water, King Fahd University of Petroleum and Minerals, Dhahran 31261, Saudi Arabia; umairbaig@kfupm.edu.sa (U.B.); wfallata@kfupm.edu.sa (W.S.F.); 2 Center for Research Excellence in Nanotechnology, King Fahd University of Petroleum and Minerals, Dhahran 31261, Saudi Arabia; abuzar@kfupm.edu.sa; 3 Department of Physics and Center for Research Excellence in Nanotechnology, King Fahd University of Petroleum and Minerals, Dhahran 31261, Saudi Arabia; makader@kfupm.edu.sa; 4 Department of Mechanical Engineering, King Fahd University of Petroleum and Minerals, Dhahran 31261, Saudi Arabia

**Keywords:** nanocomposites, pulsed laser ablation, visible-light-active photocatalyst, PEC water splitting

## Abstract

A visible-light-active nickel oxide–graphitic carbon nitride (NiO@g-CN) hetero-structured nanocomposite was synthesized for the first time by pulsed laser ablation in liquid and used as a photoanode material in photoelectrochemical water-splitting reaction with a solar simulator. It was found that the photoelectrochemical performance of PLAL synthesized NiO@g-CN nanocomposite as photoanode, compared to g-CN as photoanode showed fourfold enhancements in photocurrent density under visible light. FT-IR, XRD, FE-SEM, and EDX consistently showed the proper anchoring of nano-sized NiO on g-CN. UV-DRS and the band gap estimation showed the narrowing down of the band gap energy and consequent enhancement in the visible-light absorption, whereas photoluminescence spectroscopy confirmed the reduction of the recombination of photo-excited electron hole pairs as a result of the anchoring of NiO on g-CN. The photoelectrochemical performance of g-CN and the NiO@g-CN nanocomposite photoanodes was compared by linear sweep voltammetry (LSV), Chronoamperometry (I-t), and Electrochemical Impedance Spectroscopy (EIS). All of these results of the characterization studies account for the observed fourfold enhancement of photocurrent density of NiO@g-CN nanocomposite as photoanode in the photoelectrochemical reaction.

## 1. Introduction 

In the age of an ever-increasing need for energy and growing concern for the environment, the realization and utilization of carbon-free hydrogen fuel remain as the hope for catering to the global energy needs and mitigating the environmental damage caused by fossil fuels [1,2,3,4]. The current method widely used for hydrogen production is steam reforming of the natural gas, which cannot be a complete solution, as this method releases greenhouse gases in the atmosphere, and also the hydrogen production by electrolysis is not effective, as a lot of energy has to be expended to produce hydrogen. The level of CO_2_ in the atmosphere has already reached an alarmingly dangerous level, and if it is left unchecked, it will be very catastrophic for the environment and life on earth. The method of carbon capture and sequestration to convert CO_2_ into value added hydrocarbon is quite cumbersome and requires a huge amount of energy [5,6]. Hence, these methods of hydrogen production are not only economically profitable but also cause huge damage to the already fragile environment [7,8]. A simple photoelectrochemical method using n-type TiO_2_ (rutile) semiconductor as a photoanode and platinum as the counter electrode was successfully demonstrated by Fujishima et al., where the electrons generated as a result of the irradiation of UV light on TiO_2_ caused a flow of a photo-current to the counter electrode to photoanode, and this current was maintained even in the absence of bias voltage [9]. This pioneering work triggered many environmental and energy applications, such as photocatalytic water splitting [10], CO_2_ conversion into value added hydrocarbons [11,12], and water purifications [13,14]. The major focus for the utilization of photo-catalytic and photoelectrochemical methods has been the quest for the right materials, i.e., materials that are capable of harvesting the naturally abundant solar radiation and improve the efficiency by hampering the undesired negative reactions, like the inherent recombination of the photo-generated charge carriers. 

Although TiO_2_ has been quite a ubiquitous photocatalyst for many photocatalytic and photoelectrochemical applications, such as hydrogen production, CO_2_ reduction, and environmental remediation [15,16], its wider band-gap energy restricts its use in UV region, and in addition to this, the rapid recombination of photo-generated charge carriers is another disadvantage of this material. Besides TiO_2_, other semiconductors, like NiO, WO_3_, ZnO, CdS, and CdSe, in pure, doped, and composite forms, were developed by different groups that were aiming at improving the visible-light absorption and also reducing the charge recombination [17]. The formation of a metal semiconductor junction (Schottky junction) and a junction between two dissimilar semiconducting materials (heterojunction) shrinks the band-gap energy of the material, due to the reshuffling of the density of states, and also facilitates the spatial charge separation through the drifting of the charge carriers at the junction, due to the junction-induced electric field. These two newly evolved features in the composite materials ensure better visible-light-induced photocatalytic activity, with a reasonable enhancement of efficiency. Another factor to be considered for the applications of these materials for the photoelectrochemical water splitting, in particular, is that the conduction band energy should be more negative than the hydrogen reduction potential, in order to facilitate the photo-generated electrons to spontaneously migrate to the counter electrode, to carry out hydrogen reduction. However, in the absence of the above energy compatibility, the external bias should be used in the photoelectrochemical cell. Moreover, the electric field generated by the electrons impedes the reverse reaction, where the hydrogen and oxygen in the photochemical reaction combine to form water through an explosive reaction.

In the pursuit of finding a heterogeneous photocatalyst, metal-free polymeric graphitic carbon nitride g-CN has become the center of attraction in photocatalysis. Polymeric graphitic carbon nitride (g-CN) is a visible-light-active n-type semiconducting material with the band-gap energy of 2.7 eV. In addition to this, g-CN has other favorable features, such as an attractive electronic structure, cost-effectiveness, nontoxicity, environmental friendliness, and thermal and chemical stability [18,19,20]; hence, this material has been used for many visible-light-driven photocatalytic applications, such as water splitting and CO_2_ reduction [21]. In spite of the visible-light activity of g-CN close to the blue spectral region, the use of pure g-CN is restricted by rapid photo-generated charge recombination, small surface area, due to the characteristics two-dimensional layered structure [22,23]. In order to minimize the rapid charge recombination and harness the positive features of g-CN, modifications like doping with metal and non-metals and compositing with other co-catalyst have been tried, and these kinds of material engineering have improved the photocatalytic activity of the material to a certain extent, in different applications [24,25,26]. Particularly, compositing n-type g-CN with different p-type semiconducting materials like BiOCl, CuS, and SnS_2_ has been tried, and it was reported that the resultant p-n heterojunction facilitated the spatial charge separation and consequently resulted in the reduction of charge recombination, which was eventually manifested as the enhancement of photocatalytic efficiency. Hence, the p-n heterojunction of g-CN with any compatible p-type material is a proven method to enhance the photocatalytic activity [27,28]. 

NiO is a p-type semiconductor, showing a perfect lattice match with g-CN; it also shows a compatible band energy structure to form type-II heterojunction with g-CN, which facilitates high charge mobility across the junction. Both the conduction band (−1.95 eV) and valance band (1.35 eV) of p-type NiO are at a higher energy position than the conduction band (−0.95 eV) and the valance band (1.70 eV) of n-type g-CN; hence, upon junction formation, the electric field is directed from g-CN side to NiO side, and this field drifts the electrons from the conduction band of NiO to that of g-CN accompanied by the drifting of holes from the valance band of g-CN to that of NiO, thereby establishing a spatial separation of photo-generated charge carriers and hence hinders the spontaneous charge recombination process. This reduced charge recombination promoted by the junction formation, coupled with the intactness of visible-light activity of g-CN, ensures that NiO is a good co-catalyst with g-CN for the photocatalytic applications, and for this same reason, this composite material can be a good material for the photoanode in photoelectrochemical applications [29,30]. 

Photocatalytic and photoelectrochemical activities of the semiconductor widely depend on the shape size and morphology of the synthesized material, and these factors are decided by the physical and chemical environment that prevails during the synthesis [31,32,33,34,35]. The well-established methods of synthesis by solvothermal, hydrothermal, and photochemical reduction have some major disadvantages, like the need for heavy instrumentation and post-purification, due to the use of various chemical intermediates. In recent years, the method of pulsed laser ablation in liquid (PLAL) is commonly used for the synthesis of pure and composite nano-structured materials, as the physical and morphological characteristics materials can be controlled by the laser parameters, such as laser wavelength and pulse laser duration, and by the pH, added surfactant, and the temperature of the liquid medium used for the synthesis [16,36,37]. Moreover, unlike other conventional methods of synthesis, PLAL does not require heavy instrumentation and purification of the material after synthesis. In the PLAL method of synthesis, the plasma plume generated by the material laser interaction creates a cavitation bubble in the liquid medium, and the expansion and the collapse of the cavitation bubbles and the consequent formation of the shock wave critically contribute to the development of shape size and morphology of the synthesized nanoparticles. The complex physical change brought about by the shockwave and the cavitation bubble initiate a unique chemical reaction between the composite partners, determining the chemical characteristics of the nanocomposite.

In the present work, we employed the PLAL method of rapid synthesis of hetero-structured inorganic–organic nickel oxide–graphitic carbon nitride (NiO@g-CN) nanocomposite, and the optical, morphological, and structural characteristics of this material used out in this study proved the resultant composite material (NiO@g-CN) is visible-light active and imparts good inhibition to the recombination of photo-generated charge carriers. The synthesized g-CN and the NiO@g-CN nanocomposite were used as photoanodes in the photoelectrochemical water-splitting reaction, and it was found that the photoelectrochemical performance of PLAL-synthesized NiO@g-CN nanocomposite photoanode, compared to the g-CN photoanode, showed four-fold enhancement in photocurrent density under visible light. The PEC performance of g-CN and the NiO@g-CN nanocomposite photoanodes was compared by I-V, I-t characteristics, and EIS measurements. To the best of the knowledge, there is no report on the fast synthesis of a hetero-structured inorganic–organic NiO@g-CN nanocomposite by pulsed laser ablation in liquid technique as photoanode material for PEC water oxidation reaction. 

## 2. Experimental 

### 2.1. Reagents and Chemicals 

Nickel oxide (NiO) nanoparticles, Melamine, and other chemicals used in this study were purchased from Merck, Darmstadt, Germany. The solvents, such as acetone, ethanol, and methanol, were of high purity, and they were locally purchased. All the chemicals were used as purchased.

### 2.2. Synthesis of Graphitic Carbon Nitride

The g-CN powder was prepared by using a simple thermal pyrolysis technique. Melamine powder was used as precursor for the synthesis of g-CN. Firstly, Melamine powder was calcined for 2 h at 550 °C, in a heating furnace, at a ramp rate of 20 °C/min, and then the obtained powder, containing tri-s-triazine units, was further calcined for 2 h at 550 °C, in a heating furnace, at a ramp rate of 20 °C/min, to obtain the g-C_3_N_4_ powder. 

### 2.3. Synthesis of Nickel Oxide–Graphitic Carbon Nitride Nanocomposite

**Figure 1:**
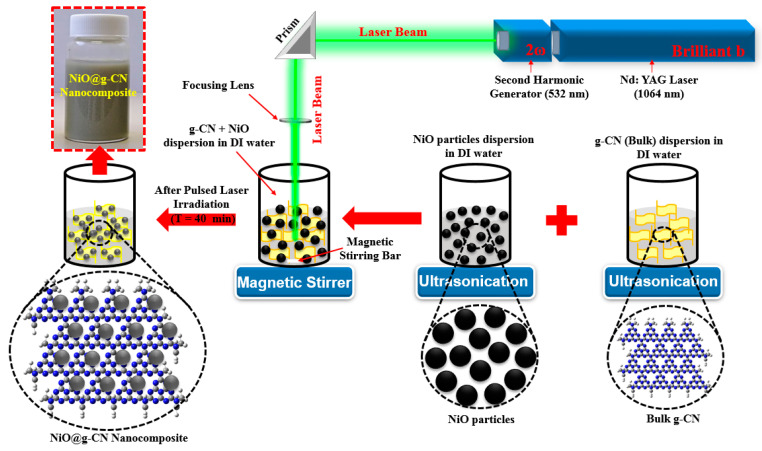
Schematic diagram for the synthesis of NiO@g-CN nanocomposite, using pulsed laser ablation in liquid approach.

A nickel oxide–graphitic carbon nitride nanocomposite (NiO@g-CN) was synthesized for the first time via pulsed laser ablation in liquid (PLAL), in the liquid medium of de-ionized water. High-energy laser radiations from the second harmonic of Q-Switched Nd:YAG laser (Brilliant B; λ = 532 nm) were used. The pulse energy of the laser beam was maintained at 300 mJ/Pulse, the pulse duration was 5 ns, and the pulse repetition was 10 Hz. The laser beam was routed to a glass beaker, where it was focused into the liquid medium, using a lens of 50 cm focal length. The mixture of g-CN and NiO was dispersed in 20 mL of de-ionized water, under ultrasonic vibrations, for 1 h, and then irradiated with a laser beam for about 40 min. In order to ensure homogeneous ablation, the mixture was stirred continuously, using a magnetic stirrer. After 40 min of irradiation, a NiO@g-CN nanocomposite solution was obtained, and it was dried at 80 °C, in an oven, for 2 h, to obtain NiO@g-CN nanocomposite powder. The graphic representation for the synthesis of NiO@g-CN nanocomposite by PLAL is shown in Figure 1**.**

### 2.4. Characterization

FE-SEM images were taken by using Filed emission electron microscope (Lyra3, Tescan, Brno, Czech Republic), equipped with an energy dispersive EDX system. TEM and HR-TEM images were taken, using a JEOL (JEM-2100F) Transmission Electron Microscope (JEOL USA Inc., Peabody, MA, USA). X-ray diffraction (XRD 6000, Shimadzu, Kyoto, Japan) with Cu-Kα radiation instrument was used for XRD studies. FT-IR spectra were obtained on a Nicolet 6700 FT-IR spectrometer IS50 (Thermo Electron Corp., Waltham, MA, USA), UV-DRS were obtained on a JASCO V-670 UV-VIS spectrophotometer (JASCO, Pfungstadt, Germany), and the PL emission spectra were acquired by Spectrofluorimeter (JASCO, FP-8500, Pfungstadt, Germany).

### 2.5. Fabrication of Photoanodes and PEC Measurements

The ink solution (2 mL) of the photocatalysts was prepared in a vial containing an ethanol-and-water mixture (1:1). For the preparation of ink, 10 mg of the photocatalyst was taken in a vial containing 2 mL of water–ethanol mixture (1:1), followed by an addition of 8 μL of Nafion^®^. The dispersion was homogenized by sonication for 1 h. The film was prepared by drop-casting the ink solution on FTO kept on hot plate (60 °C). The ink solution on FTO covered an area of 1 cm^2^. The film was left on the hot plate until it was completely dried. The electrochemical characterization of the film was carried out in a 3-electrode assembly containing 0.5 M Na_2_SO_4_ electrolyte (pH 7.1). The film on FTO served as a photoanode, while the Saturated Calomel Electrode (SCE) and Pt electrode served as reference and counter electrode, respectively. The assembly was connected to an Autolab potentiostat supported by NOVA 2.0 software (NOVA 2.0, Metrohm Autolab). A solar simulator (Oriel Sol-AAA Newport) equipped with AM-1.5G and UV cut of filters providing simulated light (1 SUN) was used as the light source. The LSV measurement was carried out by sweeping the potential from 0 to 0.7 V in dark and light conditions.

## 3. Results and Discussion

### 3.1. FT-IR Spectroscopic and XRD Analysis

**Figure 2:**
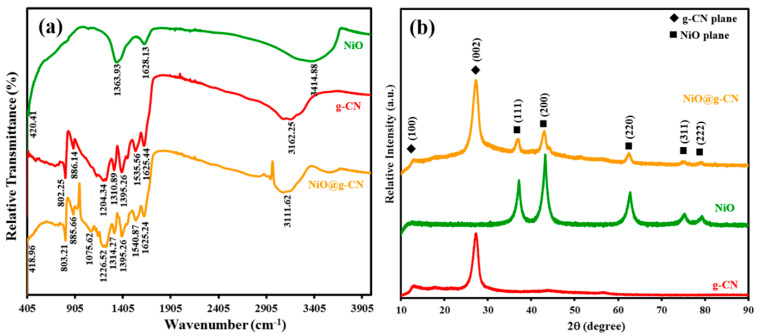
(**a**) ATR-FTIR spectra of g-CN, NiO, and the NiO@g-CN nanocomposite. (**b**) XRD patterns of pure g-CN, pure NiO, and the NiO@g-CN nanocomposite.

Figure 2a shows the comparative ATR-FTIR spectrums of g-CN, NiO, and the NiO@g-CN nanocomposite. In the FT-IR spectrum of g-CN, the absorption band at around 802.25 cm^−1^ is attributed to the breathing modes of s-triazine units. The absorption bands in the range of 1150 to 1650 cm^−1^ are attributed to the stretching vibrations of aromatic C–N heterocycles of g-CN. The strong absorption band, centered at around 3162.25 cm^−1^, was attributed to the N–H and O–H stretching vibration modes, which arises due to incomplete polycondensation of melamine and water molecules adsorbed on the g-CN surface [21]. In the FT-IR spectrum of NiO, the absorption band at 420.41 cm^-1^ was attributed to the Ni–O bond, and the absorption band at 1363.93 cm^−1^ was attributed to the carbonate ions. The absorption band at 3414.88 and 1628.13 cm^−1^ were attributed to the O–H stretching vibration and O–H deformation mode of the water molecule, respectively [38]. Meanwhile, in the FTIR spectrum of NiO@g-CN nanocomposite, the main absorption bands of g-CN and NiO were mostly shifted from lower to higher wavenumbers. This shifting of absorption bands of g-CN and NiO showed the strong interaction between g-CN and NiO and successful formation of NiO@g-CN nanocomposite, using the PLAL technique.

XRD patterns were taken to analyze the crystal structure of g-CN, NiO, and the NiO@g-CN nanocomposite. The XRD scans between 10 and 90 degrees in 2-theta scale (2θ) are shown in Figure 2b. Diffraction planes of g-CN and NiO are indicated in the XRD patterns. In the XRD pattern of g-CN, the peaks observed at around 13.0° and 27.4° were unambiguously indexed to (100) and (002) diffraction planes, respectively. The reflection (002) appeared due to interlayer-stacking of the graphitic-like aromatic structure (out-of-plane diffraction), and the reflection (100) appeared due to in-plane ordering of tri-s-triazine units (in-plane diffraction) [37]. The middle spectrum shown in Figure 2b showed the characteristic reflections of cubic ((111), (200), (220), (311), and (222)) phases of the NiO nanoparticles, as identified by JCPDS 71-1179 [38]. The XRD pattern of the NiO@g-CN nanocomposite represents the combination peaks of both individual g-CN and NiO, without any change in their positions, suggesting that the individual components of the hybrid preserve their crystal structure during the PLAL process.

### 3.2. Surface Morphology and Elemental Analysis

**Figure 3:**
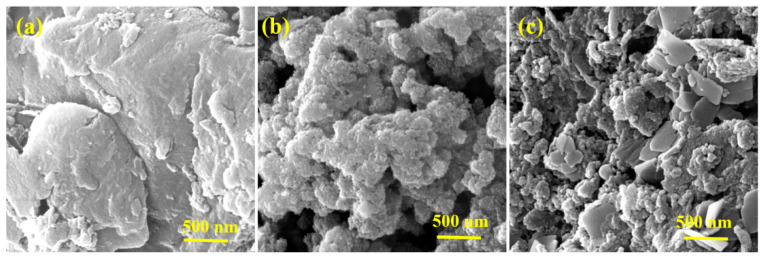
FE-SEM images of pure g-CN (**a**), pure NiO (**b**), and the NiO@g-CN nanocomposite (**c**).

The surface morphology and elemental analysis of g-CN, NiO, and the NiO@g-CN nanocomposite were evaluated by FE-SEM and FE-SEM coupled with an EDX system. The FE-SEM images of g-CN, NiO, and the NiO@g-CN nanocomposite are depicted in Figure 3. The pristine g-CN exhibits a sheet-like structure with wrinkles and folding, suggesting its thin nature (Figure 3a). On the contrary, the pure NiO shows spherical-shaped nanometer-sized particles with agglomeration and cluster formations (Figure 3b). In the FE-SEM image of the NiO@g-CN nanocomposite, the dispersion of NiO nanoparticles can be seen on g-CN sheets (Figure 3c), suggesting the formation of the NiO@g-CN nanocomposite. The thermal shock and the cavitations explosion in PLAL result in the formation of a new cationic and anionic environment on the sample surface, without modifying the basic crystal structure and phase composition. In fact, the defects in the photocatalyst are quite conducive for efficient photocatalytic activity, and this can be a good alternative for adding a dopant. In particular, anion vacancies can effectively regulate the electronic structure and energy band structure of photocatalysts, reduce atom coordination numbers, and provide more active centers, which plays an important role in improving photocatalytic efficiency.

**Figure 4:**
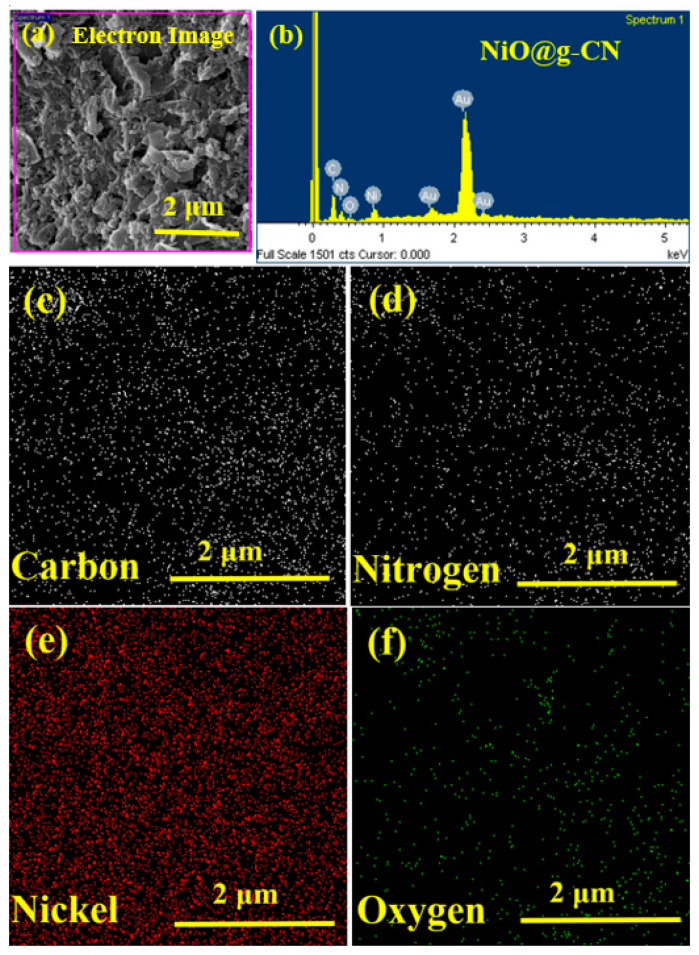
Elemental analysis of the NiO@g-CN nanocomposite. (**a**) Low-magnification FE-SEM image of the NiO@g-CN nanocomposite, showing the selected area for EDX and elemental mapping analysis. (**b**) EDX spectrum of NiO@g-CN nanocomposite. Elemental maps for carbon (**c**), nitrogen (**d**), nickel (**e**), and oxygen (**f**).

Energy-dispersive X-rays (EDX) spectroscopy was performed for the elemental analysis of the synthesized NiO@g-CN nanocomposite, as shown in Figure 4. The low-magnification FE-SEM image of the NiO@g-CN nanocomposite prepared by pulsed laser ablation, with the corresponding EDX spectrum, is shown in Figure 4a,b, respectively. The EDX spectrum (area is selected in Figure 4a) of the NiO@g-CN nanocomposite displayed peaks of C, N, O, and Ni, confirming the successful loading of NiO on g-CN sheets (Figure 4b). From corresponding elemental maps (Figure 4c–f), distribution of the elements—carbon (C), nitrogen (N), nickel (Ni), and oxygen (O)—can be seen, indicating the equal ratio of elements in the composite lattice. No peak was detected for the impurity elements in the EDX spectrum, thus proving the pure preparation of nanocomposite and its good agreement with XRD data (Figure 2b). Elemental analysis and EDX mapping images are showing the comparable results. FE-SEM, EDX mapping, and EDX elemental results altogether confirmed the successful synthesis of the NiO@g-CN nanocomposite via the PLAL route. 

**Figure 5:**
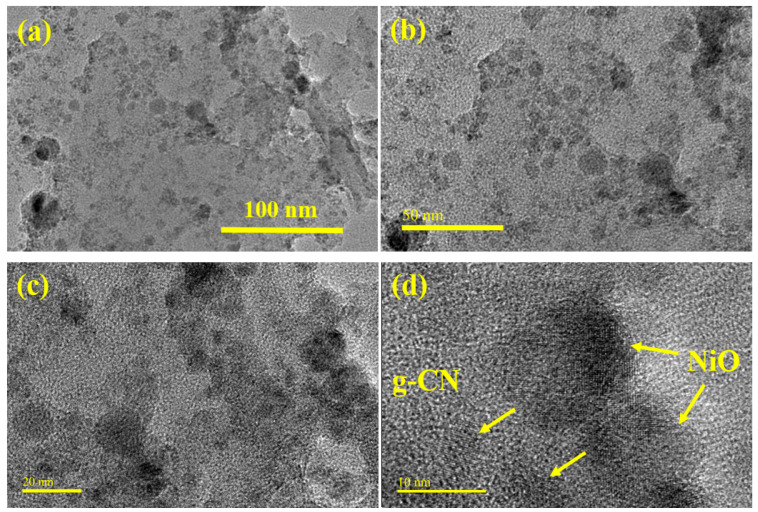
(**a**–**d**) TEM and HR-TEM images of NiO@g-CN nanocomposite synthesized via the PLAL technique, at different magnifications. NiO nanoparticles are indicated by yellow-colored arrows anchored on the g-CN surface (**d**).

The detailed morphology of the NiO@g-CN nanocomposite synthesized via the PLAL technique was evaluated by TEM and high-resolution TEM (HR-TEM) analysis. The TEM and HR-TEM images of the NiO@g-CN nanocomposite at different magnification scales are depicted in Figure 5. From TEM images of the NiO@g-CN nanocomposite (Figure 5a,b), it is clear that the NiO nanoparticles are uniformly distributed and successfully anchored on the surface of the sheet-like polymeric g-CN surface. From HR-TEM images of the NiO@g-CN nanocomposite (Figure 5c,d), it is obvious that there is close interaction between g-CN sheet and NiO nanoparticles after the formation of the NiO@g-CN nanocomposite by using the PLAL technique. HR-TEM images also demonstrate that NiO nanoparticles were closely connected with g-CN sheets, suggesting the strong interaction between the polymeric g-CN sheets and NiO nanoparticles. 

### 3.3. UV-DRS and PL Spectroscopic Analysis

**Figure 6:**
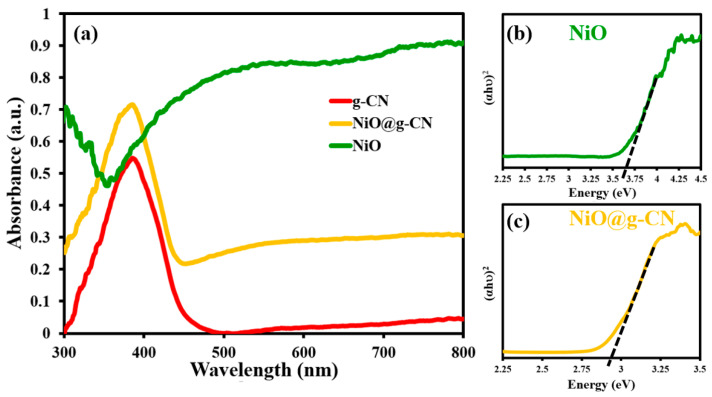
UV-DRS spectra of pure g-CN, pure NiO, and the NiO@g-CN nanocomposite (**a**). Tauc plots of NiO (**b**) and the NiO@g-CN nanocomposite (**c**).

The optical absorption properties of g-CN, NiO, and the NiO@g-CN nanocomposite were measured by UV-Vis diffuse reflectance spectroscopic (UV-DRS) analysis. The UV-DRS spectrum of g-CN in Figure 6a displays a sharp absorption peak at around 380 nm wavelength. It is due to the electronic transition of π–π* or n–π* in the tri-s-triazine ring structure of g-CN, which is in agreement with other reports [21]. However, the typical absorption of NiO is in the UV region, due to its broader band-gap energy. By anchoring NiO on the g-CN surface, we aimed to keep the absorption characteristics of g-CN intact, and at the same time forming a heterojunction between NiO and g-CN to promote the charge separation and consequent reduction of charge recombination, which is explained in the next response. Our results show that, compared to pure g-CN and NiO, the NiO@g-CN nanocomposite exhibits enhanced photo-absorption in the whole visible-light region. This can be attributed to the strong interaction between the polymeric photocatalyst g-CN and the NiO nanoparticles, which has been already suggested by other characterization results. The results of UV-DRS demonstrate that the enhanced photo-absorption in the visible-light region by nanocomposite formation of g-CN and NiO, which is good for visible-light-driven photoelectrochemical water oxidation reaction.

Tauc plots of NiO nanoparticles and the NiO@g-CN nanocomposite are depicted in Figure 6b,c. A Tauc plot of pure g-CN is depicted in Supplementary Materials Figure S1. The band-gap energies of NiO nanoparticles and the NiO@g-CN nanocomposite were estimated from the Tauc plot, by extrapolating the straight portion of (αhν)^2^ versus (hν) plot. The estimated E_g_ value of NiO nanoparticles and the NiO@g-CN nanocomposite by extrapolating the straight portion of (αhν)^2^ versus (hν) plot are ~3.6 eV and ~2.85 eV, respectively, as shown in Figure 6b,c. The presence of the g-CN in the NiO@g-CN nanocomposite has proved effectiveness in shifting the band gap of NiO nanoparticles toward the visible-light range of the solar-spectrum. The decreased bandgap of NiO@g-CN nanocomposite means that lower-energy radiation can stimulate the photocatalysts to generate electron–hole pairs. Therefore, the better photocatalytic performance of the NiO@g-CN nanocomposite can be expected by longer-wavelength electromagnetic radiation.

**Figure 7:**
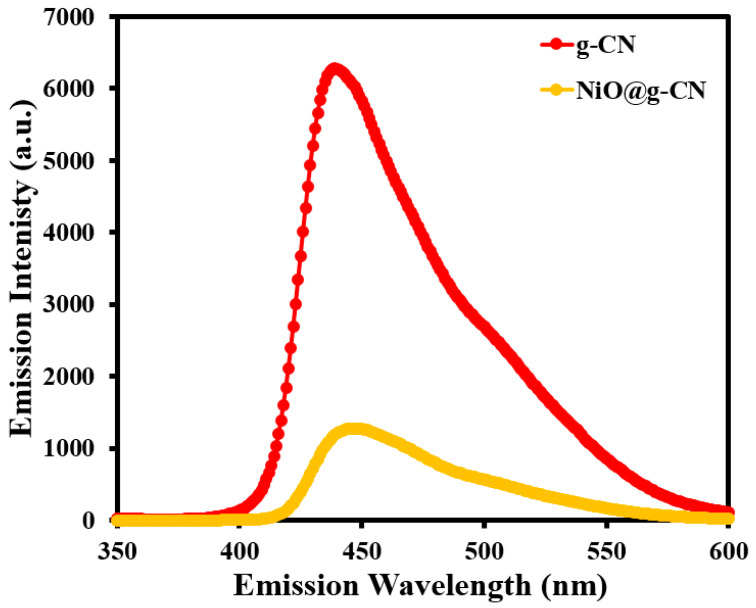
Photoluminescence spectra of g-CN and the NiO@g-CN nanocomposite.

The electron–hole recombination behavior of the g-CN and NiO@g-CN nanocomposite photocatalysts was examined by photoluminescence spectroscopy at an excitation wavelength of 320 nm. Figure 7 shows the PL spectra of g-CN and the NiO@g-CN nanocomposite photocatalysts from 350 to 600 nm emission-wavelength range. For both g-CN and the NiO@g-CN nanocomposite photocatalysts, the emission band was centered at ~450 nm. However, the emission intensity of the pristine g-CN after anchoring of NiO nanoparticles via the PLAL technique was effectively decreased, demonstrating the reduced electron–hole recombination rate and enhanced photocatalytic activity of the NiO@g-CN nanocomposite photocatalyst. PL emission in g-CN is due to the photo-generated electron–hole recombination that takes place, when the electrons in the conduction band transfer to the valance band. When NiO is anchored on the polymeric surface of g-CN, this results in the formation of a p-n heterojunction between the p-type NiO and n-type g-CN semiconductors, which promotes the charge separation due to the electric field in the junction and consequently results in the reduction of charge recombination. This reduced-charge recombination is reflected as a diminished PL-peak intensity that is quite evident in our PL spectrum in Figure 7.

### 3.4. Photoelectrochemical Measurements

**Figure 8:**
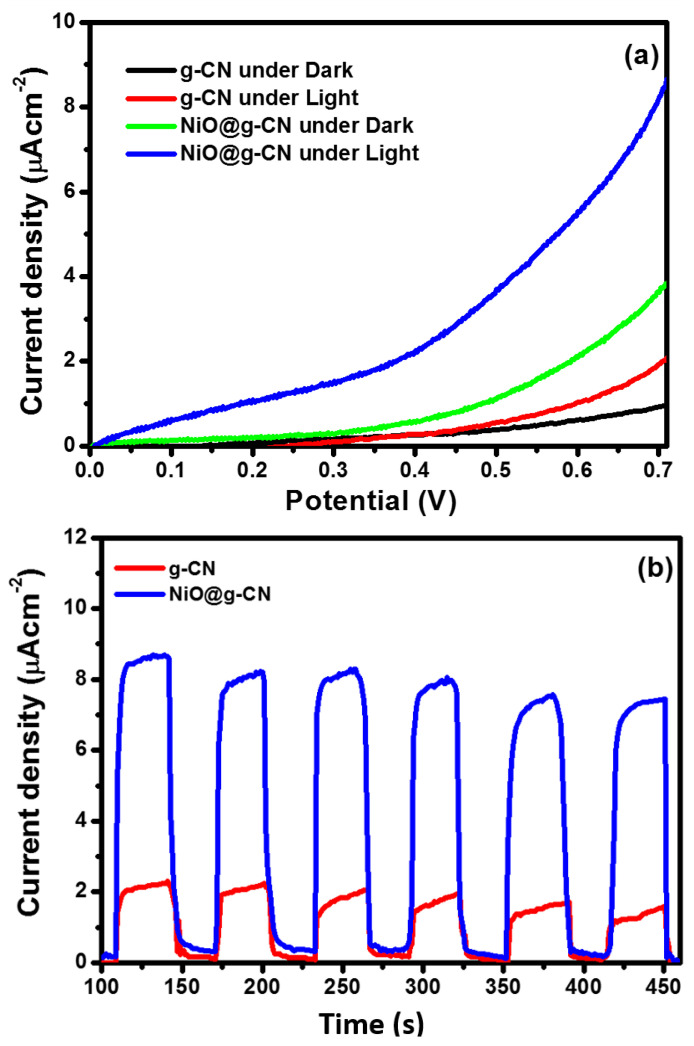
Linear-sweep voltammetric response of g-CN and NiO@g-CN nanocomposite photoanodes under dark and visible-light irradiation (**a**). Chronoamperometric photocurrent–time responses of g-CN and NiO@g-CN nanocomposite photoanodes under periodically chopped visible-light irradiations (light on/off cycle) (**b**).

The electrodes of g-CN and the NiO@g-CN nanocomposite photocatalyst were fabricated and were used as photoanodes, to study the photoelectrochemical (PEC) water-splitting performance under simulated one-sun-light irradiation. The PEC water-splitting performance of the g-CN and NiO@g-CN nanocomposite photoanodes was evaluated by measuring the current density in LSV measurements under dark and light exposure. The comparative I-V characteristics of g-CN and NiO@g-CN nanocomposite photoanodes under dark and light irradiations are shown in Figure 8a. It is clear from the I-V characteristics that the magnitude of photocurrent density increased with increasing the voltage sweep for g-CN and NiO@g-CN nanocomposite photoanodes under dark and light irradiations. Upon exposure to light, the current density of the g-CN photoanode was increased from ~0.92 μA/cm^2^ (dark current) to ~2.0 μA/cm^2^, at 0.7 volt, versus SCE. Similarly, on exposure to visible light, the current density of the NiO@g-CN nanocomposite photoanode was increased from ~3.72 μA/cm^2^ (dark current) to ~8.65 μA/cm^2^, at 0.7 volt, versus SCE. The enhancement in the current densities of g-CN and the NiO@g-CN nanocomposite photoanodes after exposure to light demonstrates the photo-responsive feature of the synthesized catalysts. The findings of I-V characteristics for PEC water-splitting demonstrate that, as compared to the g-CN photoanode, the NiO@g-CN nanocomposite photoanode showed a four-fold enhancement in the photocurrent density under visible-light irradiations and thus improved PEC performance. The lower photocurrent response of pristine g-CN could be attributed to high photo-generated charge recombination in g-CN and the improved photocurrent response of NiO@g-CN nanocomposite photoanode produced by reduced photo-generated charge recombination in NiO@g-CN nanocomposite by anchoring of NiO nanoparticles in the g-CN sheets via the PLAL technique, which is supported by PL results in Figure 7.

The photocurrent density of g-CN and the NiO@g-CN nanocomposite photoanodes was also measured under a chopped light-irradiation condition (light on/off cycle), at a constant 0.7-volt bias voltage. Chronoamperometric photocurrent–time responses of g-CN and the NiO@g-CN nanocomposite photoanodes under periodically chopped visible-light irradiations (light on/off cycle) are depicted in Figure 8b. The current density was measured for ~350 s, with 30 s of intervals at an applied bias of 0.7-volt, vs. SCE. The result indicated that, upon exposure to visible light, the photocurrent generated instantaneously and reached a steady state and fell sharply as soon as the light was switched off. There was an initial slight decrease in the photocurrent, which became stable with time, indicating good stability of photoanodes under visible-light irradiation and constant applied potential. 

**Figure 9:**
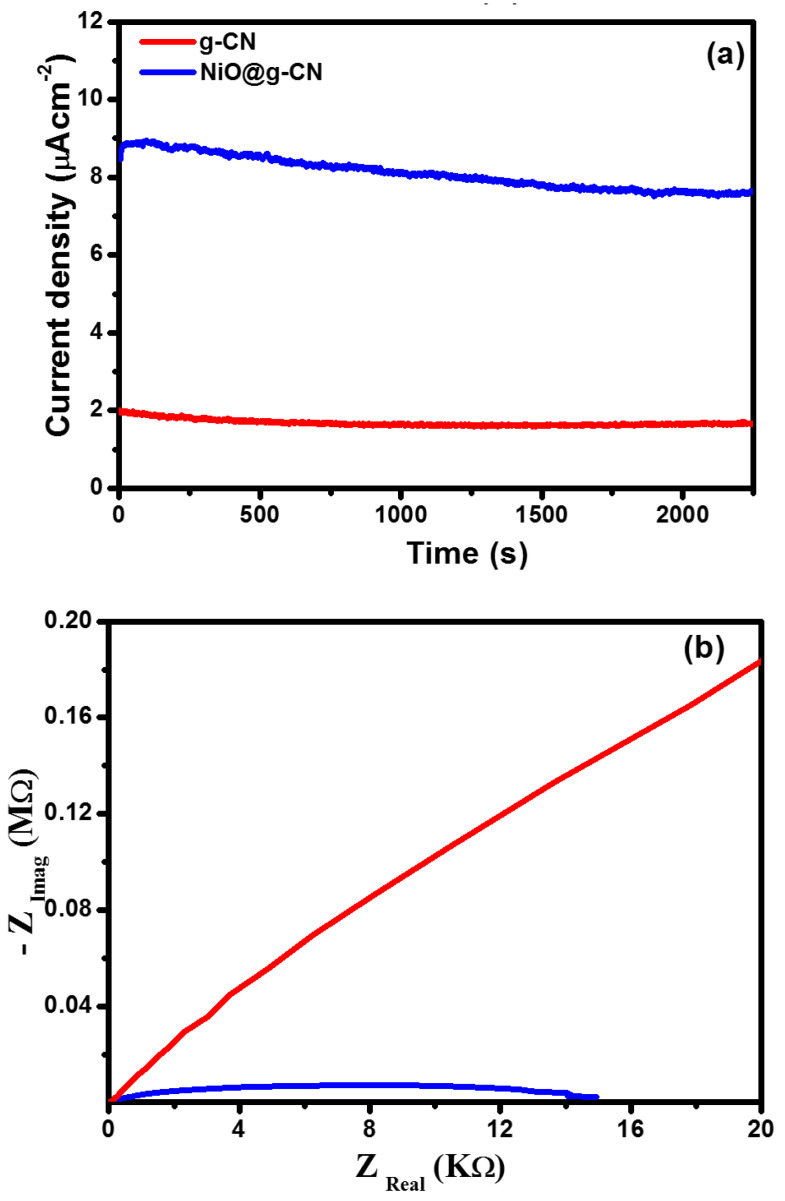
Chronoamperometric stability testing of g-CN and the NiO@g-CN nanocomposite photoanodes under continuous visible-light irradiations (**a**). Nyquist plots of g-CN and NiO@g-CN nanocomposite photoanodes (**b**).

The photocurrent density of g-CN and NiO@g-CN nanocomposite photoanodes was also measured under continuous visible-light irradiations condition, at a constant 0.7-volt bias voltage vs. SCE, to measure the stability of the fabricated photoanodes in the PEC water-splitting experiment. Chronoamperometric stability testing of g-CN and NiO@g-CN nanocomposite photoanodes in the PEC water-splitting experiment under continuous visible-light irradiations is shown in Figure 9a. After 2250 s of continuous visible-light irradiations, the photocurrent density of g-CN and NiO@g-CN nanocomposite photoanodes was slightly decreased, and that decrease can be attributed to photo-corrosion of the fabricated photoanodes in the electrolyte solution. 

The charge-transfer behavior in g-CN and NiO@g-CN nanocomposite photoanodes was evaluated by EIS (electrochemical impedance spectroscopy) measurements. Figure 9b shows the Nyquist plots of g-CN and NiO@g-CN nanocomposite photoanodes. Generally, the smallest radius of the arc denotes more effective separation and transport of photo-generated charge carriers. In Figure 9b, NiO@g-CN nanocomposite photoanode displays the smallest radius of the arc compared with the pristine g-CN photoanode, suggesting the fastest interfacial charge transport process and lowest charge-transfer resistance of photo-generated charge carriers in the NiO@g-CN nanocomposite photoanode.

**Figure 10:**
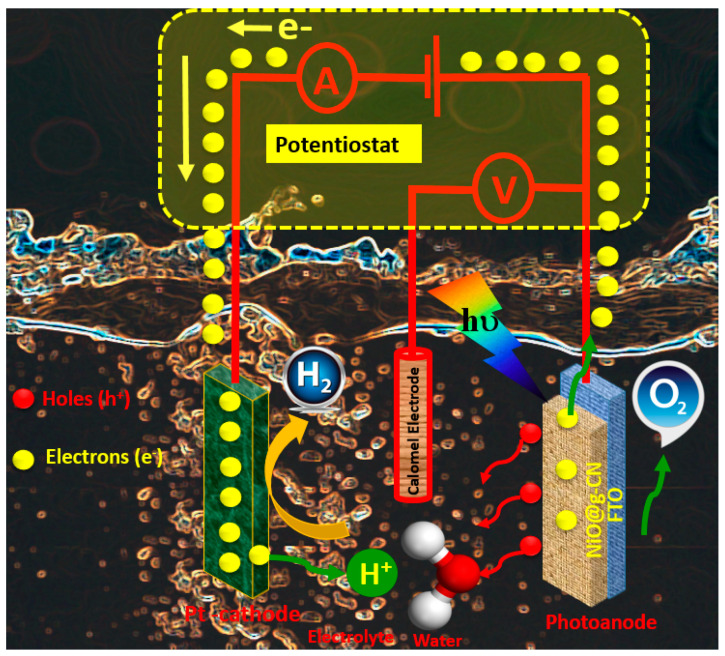
Schematic sketch of proposed photoelectrochemical working mechanism of the NiO@g-CN nanocomposite photoanode for water splitting.

Based on the PEC measurements, a schematic sketch of the photoelectrochemical working mechanism of NiO@g-CN nanocomposite photoanode for the water-splitting experiment is presented in Figure 10, showing the photo-oxidation and reduction processes taking place during the reaction, where the NiO@g-CN nanocomposite is used as a photoanode under visible-light irradiations.

## 4. Conclusions

A visible-light-active NiO@g-CN nanocomposite was synthesized by pulsed laser ablation in liquid, using a focused beam of 532 nm pulsed laser source. The morphological, structural, and elemental analyses carried out in this work confirmed the anchoring of NiO nanoparticles on g-CN sheets. The first effect of this nanocomposite formation (anchoring of NiO nanoparticles on g-CN sheets) is manifested as enhanced visible-light absorption of NiO@g-CN in the absorption spectra deduced from diffuse reflectance spectra, and the second effect is manifested as reduced electron hole recombination observed as the reduction of photoluminescence intensity of NiO@g-CN in the PL spectra. The improved material characteristics in NiO@g-CN culminated in four-fold higher photocurrent generated by the NiO@g-CN photo anode compared to the anchoring of NiO nanoparticles on g-CN sheets anchoring of NiO nanoparticles on g-CN sheets by pure g-CN photoanode.

## Supplementary Materials

The following are available online at https://www.mdpi.com/2079-4991/10/6/1098/s1, Figure S1: Tauc’s plot of g-CN.
