# Supplementary material for: Laser Induced Anchoring of Nickel Oxide Nanoparticles on Polymeric Graphitic Carbon Nitride Sheets Using Pulsed Laser Ablation for Efficient Water Splitting under Visible Light
Source: Nanomaterials (Basel). 2020 Jun 2;10(6):1098. doi: 10.3390/nano10061098 (PMC7353223; doi:10.3390/nano10061098)
Supplement: Supplementary file 1 [file nanomaterials-10-01098-s001.pdf]

Supplemnrty Information File

Laser Induced Anchoring of Nickel Oxide Nanoparticles on Polymeric Graphitic Carbon Nitride Sheets Using Pulsed Laser Ablation for Efficient Water Splitting under Visible Light

Umair Baig [1], A. Khan [2], M.A. Gondal [3,*], M.A. Dastageer [3] and W.S. Falath [1,4]

[1] Center of Research Excellence in Desalination & Water Treatment and Center for Environment and Water, King Fahd University of Petroleum and Minerals, Dhahran 31261, Saudi Arabia; umairbaig@kfupm.edu.sa (U.B.); wfallata@kfupm.edu.sa (W.S.F.)

[2] Center for Research Excellence in Nanotechnology, King Fahd University of Petroleum and Minerals, Dhahran 31261, Saudi Arabia; abuzar@kfupm.edu.sa

[3] Department of Physics and Center for Research Excellence in Nanotechnology, King Fahd University of Petroleum and Minerals, Dhahran 31261, Saudi Arabia; makader@kfupm.edu.sa

[4] Department of Mechanical Engineering, King Fahd University of Petroleum and Minerals, Dhahran 31261, Saudi Arabia

* Correspondence: magondal@kfupm.edu.sa; Tel.: +96-6386-02351 or +96-6386-03274

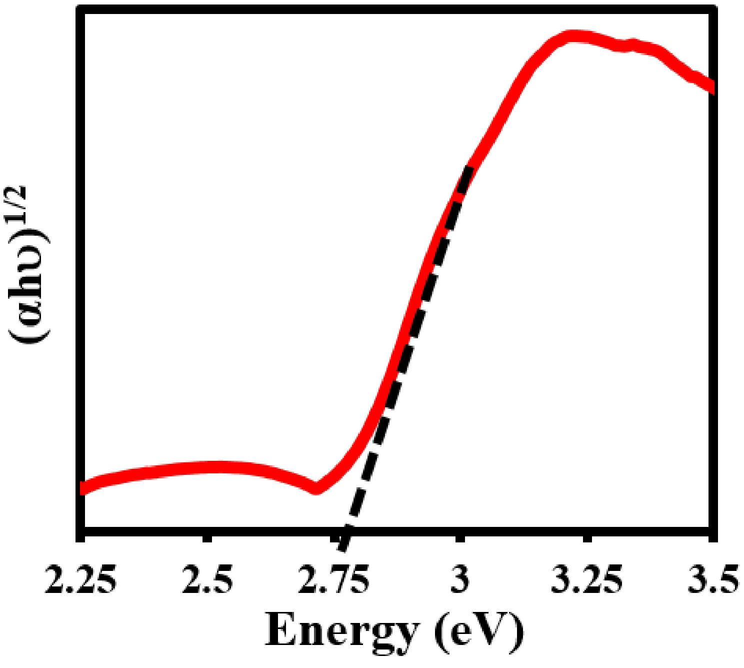

Figure S1. Tauc's plot of g-CN.
